# MiR-4701-3p predicts prognosis of non-small cell lung cancer and regulates cell biological behaviors

**DOI:** 10.1186/s41065-025-00612-3

**Published:** 2025-11-28

**Authors:** Zhenhua Hao, Yitao Sun, Guangfu Xu, Jiangtao Cao, Yang Pan

**Affiliations:** 1 Department of Cardiothoracic Surgery, Huashan Hospital, Fudan University, Shanghai, 200040 China; 2 Department of Cardiothoracic Surgery, Kunming Medical University Second Affiliated Hospital, Kunming, 650101 China; 3 Department of Pathology, Zhejiang Provincial People’s Hospital, Hangzhou, 310014 China; 4 Department of Respiratory and Critical Care Medicine, First Medical Center of Chinese PLA General Hospital, Beijing, 100853 China; 5 Department of Respiratory and Critical Care Medicine, The Affiliated Hospital of Youjiang Medical University for Nationalities, No.18, Zhongshan 2nd Road, Youjiang District, Baise City, Guangxi 533000 China; 6 Key Laboratory of Research and Development on Clinical Molecular Diagnosis for High-Incidence Diseases of Baise, Guangxi, 533000 China

**Keywords:** MiR-4701-3p, Non-small cell lung cancer, Prognostic, Tumor progression, Cell function

## Abstract

**Background & objectives:**

Non-small cell lung cancer (NSCLC) represents a malignant neoplasm exhibiting high incidence and mortality rates, marked by diminished patient survival probabilities and an unfavorable prognosis. The primary objective of this research is to investigate the prognostic value of miR-4701-3p in NSCLC and its regulatory role in tumor progression.

**Methods:**

In this study, a cohort comprising 105 patients with NSCLC was enrolled. Expression levels of miR-4701-3p in tissues and cell lines were assayed using RT-qPCR. To investigate the correlation between miR-4701-3p and clinicopathological features, the χ2 test was applied. Additionally, Kaplan-Meier survival analysis alongside Cox regression modeling were performed to evaluate the prognostic significance of miR-4701-3p.

**Results:**

miR-4701-3p was markedly upregulated in NSCLC tissues and cells. miR-4701-3p was significantly correlated with TNM stage and lymph node metastasis (LNM) in NSCLC patients. The group exhibiting high miR-4701-3p expression demonstrated a markedly decreased 5-year survival rate in comparison to the low expression group. According to Cox regression analysis, miR-4701-3p expression, TNM stage, and lymph node metastasis (LNM) emerged as independent predictors of the 5-year survival rate among NSCLC patients. By downregulating miR-4701-3p, a substantial inhibition of NSCLC cell proliferation, migration, and invasion was observed, accompanied by enhanced apoptotic activity. Conversely, overexpressing miR-4701-3p can enhance cell proliferation, invasion, and migration abilities while inhibiting cell apoptosis.

**Conclusion:**

The elevated expression of miR-4701-3p in NSCLC tissues has certain predictive value for poor prognosis in patients and may be involved in malignant behaviors of NSCLC cells.

## Introduction

Lung cancer remains one of the most prevalent malignancies in China and worldwide, with its incidence continuing to rise and ranking first among all cancers [1]. Lung cancer can be classified in various ways. The most common pathological classification broadly divides it into non-small cell lung cancer (NSCLC) and its counterpart, small cell lung cancer (SCLC). Among them, NSCLC comprises roughly 85% of all lung cancer instances [2]. The adoption of stage-specific treatment regimens currently represents the most effective clinical strategy [3]. Despite continuous efforts to develop personalized therapies aimed at improving patient outcomes, the five-year survival rate for lung cancer remains below 20% [4]. This persistently poor prognosis poses a major clinical challenge. Therefore, molecular-level research may provide new insights into identifying reliable biomarkers for predicting long-term outcomes in patients with lung cancer.

MicroRNAs (miRNAs) are small non-coding RNAs approximately 22–25 nucleotides in length that post-transcriptionally regulate gene expression [5, 6]. Aberrant miRNA expression has been implicated in the initiation and progression of various human malignancies, including lung cancer [7]. Dysregulated miRNAs can influence key cellular processes such as proliferation, apoptosis, and differentiation, thereby serving as potential diagnostic or prognostic biomarkers in cancer [8].

As a potential key factor, the expression level of miR-4701-3p in tumors and its regulatory role in tumor progression have attracted much attention. Microarray data analysis has reported that miR-4701-3p is upregulated in the peripheral blood of lung cancer patients [9]. miR-4701-3p promotes proliferation in colon cancer cells, suggesting its involvement in tumorigenesis [10]. Moreover, its closely related strand, miR-4701-5p, has also been found elevated in breast cancer cells, further highlighting the potential functional relevance of the miR-4701 locus in cancer biology [11]. However, despite these findings, the role of miR-4701-3p in NSCLC remains largely unexplored. In particular, its expression pattern, prognostic significance, and molecular mechanisms in NSCLC progression have not been elucidated. Therefore, this study aimed to investigate the clinical relevance and biological function of miR-4701-3p in NSCLC, to clarify whether it could serve as a novel biomarker or therapeutic target for this disease.

## Materials and methods

### Study subjects

A total of 105 NSCLC patients hospitalized in First Medical Center of Chinese PLA General Hospital from December 2018 to December 2019 were selected, including 74 males and 41 females, aged 37 to 71 years. Inclusion criteria were: (1) no prior radiotherapy, chemotherapy, or surgical treatment before admission; (2) assessable lesions; (3) confirmed diagnosis by pathological examination; and (4) eligibility for surgical treatment. Exclusion criteria were: (1) coexisting malignancies in other sites; (2) severe liver, kidney, or cardiovascular diseases; (3) mental abnormalities; and (4) incomplete clinical data. The Ethics Committee of First Medical Center of Chinese PLA General Hospital granted approval for this study, and informed consent was obtained from all participating patients.

### Clinical data collection and tissue sample acquisition

Data pertaining to all patients’ clinical characteristics were gathered, encompassing demographics such as age and gender, along with smoking and drinking histories. Information on tumor type, histological grade of differentiation, tumor dimensions, TNM staging, and presence of lymph node metastasis (LNM) was also recorded. Specimens of cancerous tissue and adjacent non-cancerous tissue (situated more than 2 cm from the tumor margin) were procured from all patients. Upon acquisition, these samples were promptly immersed in liquid nitrogen and subsequently transferred to a −80 °C freezer for preservation, destined for subsequent real-time fluorescent quantitative polymerase chain reaction (qPCR) analysis.

### Cell culture

The four NSCLC cell lines, A549, H460, H1299, and MES-1, were purchased from Procell Life Science & Technology Co., Ltd. in Wuhan. The human normal lung epithelial cell line, specifically BEAS-2B, was acquired from the Shanghai Cell Bank of the Chinese Academy of Sciences and maintained in RPMI-1640 medium (Gibco, USA) supplemented with 10% fetal bovine serum (Gibco). If contamination or infection was observed in the cells, 100 U/ml penicillin and 100 mg/ml streptomycin (double antibiotics; Gibco) could be added to the medium as appropriate to eliminate the infection source. Cells were all maintained in a 37 °C incubator with an atmosphere of 5% CO_2_.

### Cell transfection

Cells in the logarithmic growth phase were plated in 6-well plates (at a concentration of 1 × 10^6^ cells/mL) before transfection to ensure a cell confluence of over 50% at the time of transient transfection. According to the protocol of the Lipofectamine 3000 reagent kit (supplied by Thermo Fisher Scientific, USA), human cells were transfected with miR-4701-3p inhibitor (ACACCACACCCAUCACCCAU), miR-4701-3p mimics (AUGGGUGAUGGGUGUGGUGU), or their NC negative controls (NC), respectively. Transfection materials were synthesized by GenePharma (Shanghai, China). Transfection was performed using Lipofectamine 3000 at a final concentration of 2.5 µL/mL, with miRNA mimics or inhibitors at a final concentration of 50 nM, following the manufacturer’s instructions. After 48 h of transfection, the transfection efficiency was assessed, and subsequent experiments were conducted.

### CCK-8 assay

After the transfection process, the cells were distributed into 96-well microplates (Corning, USA), with each well containing a density of 5 × 10³ cells. CCK-8 reagent (Dojindo, Kumamoto, Japan) was added to each well at 0 h, 24 h, 48 h, and 72 h. And the plates were then incubated in an environment of 37 °C and 5% CO_2_ for a duration. Subsequently, a microplate spectrophotometer was employed to measure the optical density (OD) at a specific wavelength of 450 nm.

### Transwell migration assay

To assess cell migration activity, 24 h after transfection, cells were plated into Transwell chambers (8 μm pore size; Corning) at a density of 4 × 10^5^ cells per well in a serum-free medium. The lower chamber was supplemented with a full medium. After 24 h of incubation, the chambers were removed, and non-migrated cells were removed. Subsequently, the cells underwent fixation with paraformaldehyde (Sigma, USA), were stained using crystal violet solution (Beyotime, China), and were then photographed. The purple areas were scanned using ImageJ software, and the results were statistically analyzed with three replicates.

### Transwell invasion assay

To evaluate cell invasion activity, cells were cultured for 24 h and then plated into Transwell chambers (8 μm pore size, pre-coated with Matrigel matrix gel (BD Biosciences, USA) 24 h prior) at a density of 8 × 10^5^ cells per well in serum-free medium. The lower chamber was supplemented with a complete medium. Following a 24-hour incubation period, the chambers were detached and the non-migrated cells were discarded. Subsequently, the remaining cells were fixed using paraformaldehyde, stained with a crystal violet stain, and then photographed. The purple areas were scanned using ImageJ software, and the results were statistically analyzed with three replicates.

### Apoptosis assay (flow cytometry)

Apoptosis in the cells was identified utilizing a cell apoptosis assay kit (BD Biosciences, USA). Cells were collected 48 h after transfection, washed three times with PBS, and resuspended in an appropriate amount of binding buffer. After staining with 1 µL of annexin-V fluorescein isothiocyanate (FITC; Beyotime) in the dark for 15 min, 1 µL of propidium iodide (PI) was added. Finally, the apoptosis rate was detected using a flow cytometer.

### RNA extraction and RT-qPCR

Total RNA was isolated from tissues and cells employing TRIzol reagent (Merck, Germany), followed by quantification and quality assessment using a NanoDrop spectrophotometer. This RNA was then reverse-transcribed to cDNA using the PrimeScript™ RT reagent Kit from Takara Bio (Kusatsu, Japan). The conditions for performing the reverse transcription process were as follows: an initial step at 42 °C for 2 min, followed by 15 min at 37 °C, and a final step at 85 °C for 5 s. A clean 96-well PCR plate was used, with each well containing a mixture of 2 µL of cDNA and 18 µL of SYBR Green PCR Master Mix (Takara Bio, Kusatsu, Japan), totaling 20 µL for RT-qPCR. The internal control gene employed was U6, and the relative gene expression levels were determined by applying the 2^-ΔΔCt^ method.

### Follow-up

All patients were followed for 5 years after surgery through outpatient visits, telephone interviews, and WeChat communication. Overall survival was defined as the time from surgery to death, with death serving as the survival endpoint. Patients who were still alive at the last follow-up (December 2024) were censored at that date. If the exact time of the endpoint event could not be determined, the case was recorded as right-censored. The follow-up period ended in December 2024. Clinical information, including recurrence and survival status, was recorded at each follow-up. Poor prognosis was defined as postoperative recurrence or all-cause mortality, whereas patients without these events were classified as having a good prognosis.

### Statistical methods

Statistical analyses were performed using SPSS 25.0, while graphical illustrations were created with GraphPad Prism 9. All experiments were independently repeated at least three times (biological triplicates), and data are presented as mean ± standard deviation (SD). Comparisons between two groups were carried out using the independent-samples t-test. For count data, presentations were made in terms of cases or percentages, and comparisons among groups were performed using the χ² test. Pearson’s correlation coefficient was applied to assess the relationship between miR-4701-3p and the clinicopathological characteristics of NSCLC. Survival analysis of NSCLC patients was conducted using Kaplan-Meier curves. Cox proportional hazards regression analysis was utilized to identify risk factors influencing the prognosis of lung cancer patients. The clinical variables included in the cox regression analysis are listed in Table 1. The power analysis was conducted using a medium effect size (d = 0.5), a significance level of 0.05, and a desired power of 0.85 (two-tailed). The clinical sample size included meets the requirements of the power analysis. A statistical significance level was set at *P* < 0.05.

**Table 1:** Correlation between miR-4701-3p expression levels and clinical features of NSCLC patients

Variables	No. (*n* = 105)	miR-4701-3p expression			*P* value
		Low (*n* = 58)		High (*n* = 47)	
Age (years)					0.053
≤ 50	39	26		13	
> 50	66	32		34	
Gender					0.436
Male	69	39		30	
Female	36	19		17	
Smoking history					0.366
No	41	24		17	
Yes	64	34		30	
Drinking history					0.366
No	41		24	17	
Yes	64	34		30	
Histology					0.088
Adenocarcinoma	73	44		29	
Squamous cell carcinoma	32	14		18	
Differentiation					0.226
High and moderate	87	50		37	
Poor	18	8		10	
Tumor size					0.151
< 3	84	49		35	
≥ 3	21	9		12	
TNM stage					0.001
I	85	57		28	
II-III	20	1		19	
LNM					0.014
Negative	92	55		37	
Positive	13	3		10	

## Results

### Expression of miR-4701-3p in NSCLC and adjacent normal tissues

**Fig. 1:**
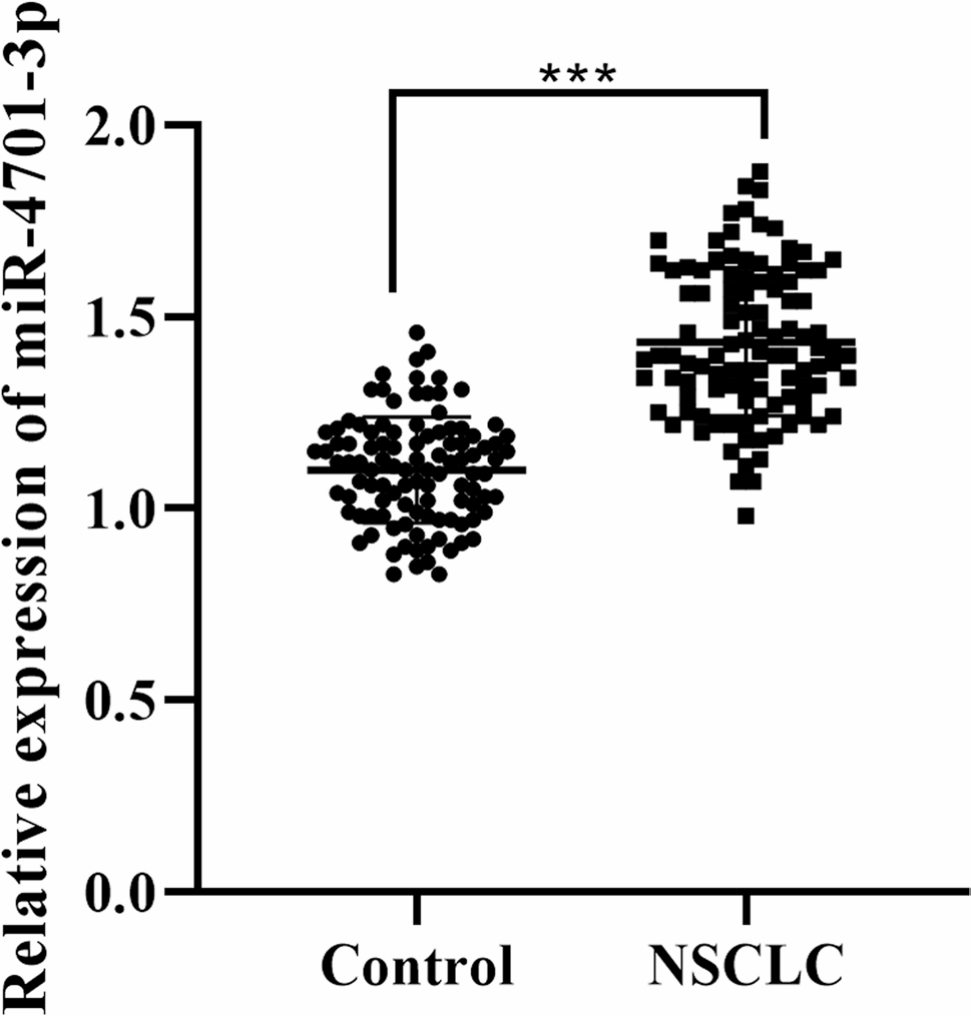
Expression of miR-4701-3p in NSCLC and adjacent non-cancerous tissues (*n* = 105). Data are presented as mean ± sd. Statistical significance was determined using Student’s t-test. ***P < 0.01

Compared with adjacent normal tissues, miR-4701-3p in NSCLC tissues was significantly increased (Fig. 1, *P* < 0.01).

### Relationship between miR-4701-3p and clinicopathological characteristics of NSCLC

Considering the average relative expression level of miR-4701-3p in NSCLC tissues, which was 1.43, 105 patients with NSCLC were categorized into two groups: a low-expression cohort and a high-expression cohort. miR-4701-3p was not associated with patient age, gender, smoking history, drinking history, histology, differentiation, or tumor size (Table 1, *P* > 0.05). Nonetheless, a notable correlation was observed between it and the TNM stage (*P* = 0.001), as well as with LNM (*P* = 0.014) among patients with NSCLC, with statistical significance (*P* < 0.05).

### Prognostic value of miR-4701-3p

**Fig. 2:**
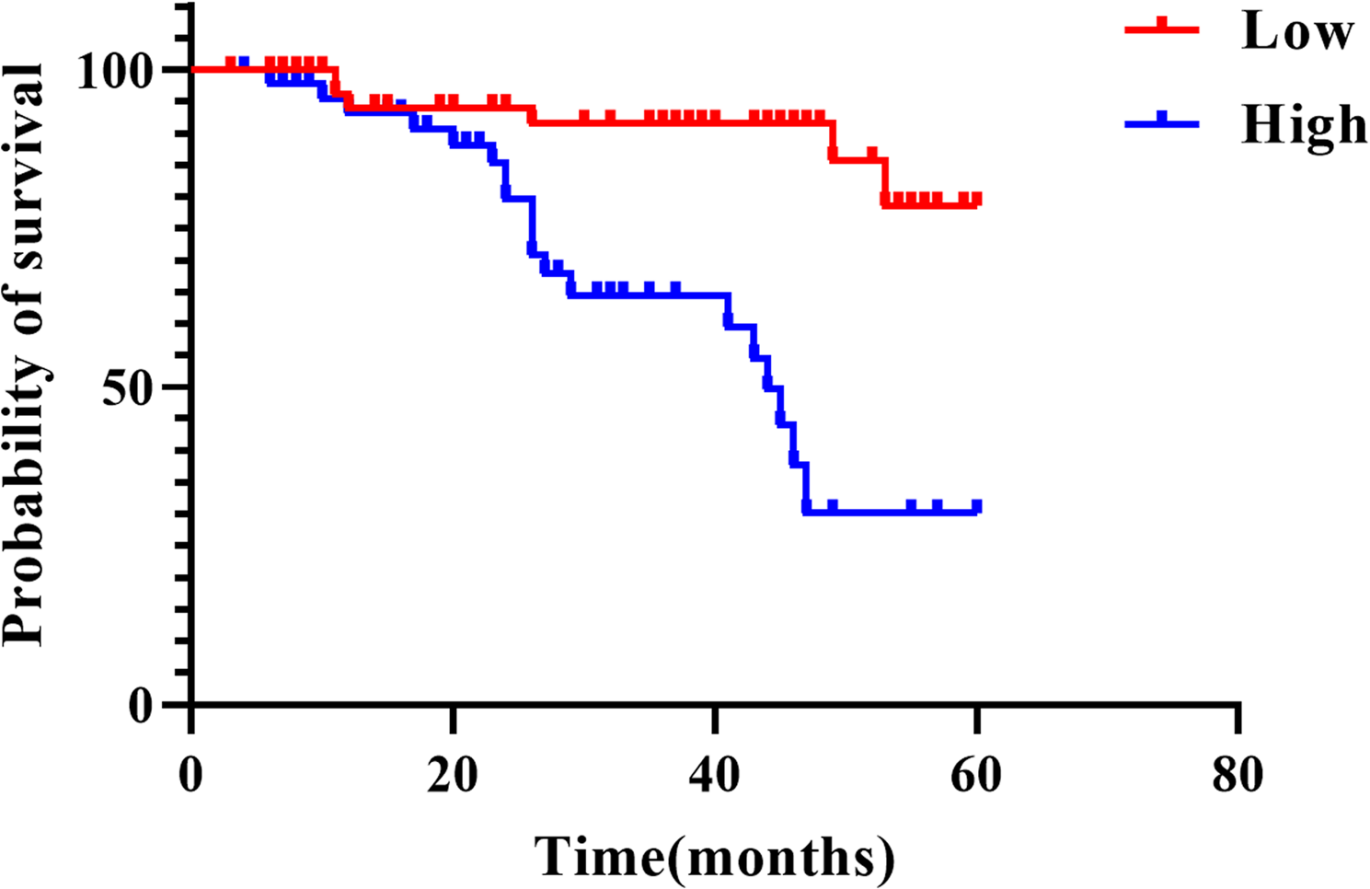
Kaplan-Meier survival curves for NSCLC patients (n = 105). Statistical analysis was performed using the log-rank test. ***P<0.01

Kaplan-Meier survival analysis revealed a statistically significant decrease in the five-year survival rate among patients exhibiting high miR-4701-3p expression, compared to those with low expression levels (Fig. 2, *P* < 0.01).

### Risk factors affecting the five-year survival rate of NSCLC patients

Cox regression analysis revealed that TNM stage (HR = 6.107, 95%CI = 3.020–2.007, *P* < 0.001), LNM (HR = 3.314, 95%CI = 1.537–7.143, *P* = 0.002) and miR-4701-3p (HR = 8.712, 95%CI = 3.551–21.290, *P* < 0.001) were independent risk factors affecting the five-year survival rate of NSCLC patients (Table 2, *P* < 0.05).

**Table 2:** Cox regression analysis to evaluate clinical characteristic indicators

Variables	HR	95% CI	*P*-value
Age	1.069	00.530–2.151	0.853
Gender	0.992	0.480–2.049	0.984
Smoking history	1.315	0.625–2.766	0.470
Drinking history	1.315	0.625–2.766	0.470
Histology	1.267	0.622–2.580	0.514
Differentiation	1.541	0.689–3.442	0.292
Tumor size	0.869	0.376–2.007	0.743
TNM	6.107	3.020–12.350	< 0.001
LNM	3.314	1.537–7.143	0.002
miR-4701-3p	8.712	3.551–21.290	< 0.001

### The expression of miR-4701-3p in cells

**Fig. 3:**
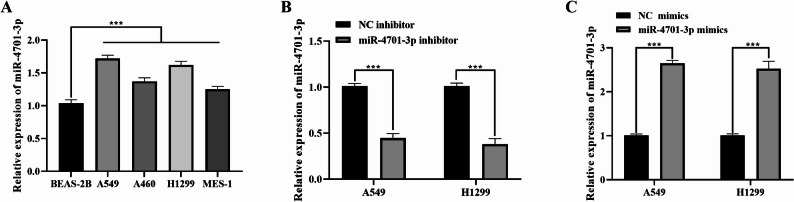
The expression of miR-4701-3p in cells (n = 3). **A** The expression levels of miR-4701-3p in various cell lines. **B** Expression in A549 and H1299 cells for the NC inhibitor group and the miR-4701-3p inhibitor group.** C** Expression in A549 and H1299 cells for the NC mimics group and the miR-4701-3p mimics group. Data are presented as mean ± sd. Statistical significance was analyzed using Student’s t-test. ***P<0.01

Compared with the normal cell line BEAS-2B, miR-4701-3p was significantly upregulated in NSCLC cell lines (*P* < 0.01, Fig. 3A), with the most pronounced difference observed in A549 and H1299 cells. Therefore, A549 and H1299 cells were selected for subsequent experiments. After inhibiting miR-4701-3p, miR-4701-3p in A549 and H1299 cells was significantly reduced (*P* < 0.01, Fig. 3B). Conversely, overexpressing miR-4701-3p resulted in a significant increase in its expression level in both A549 and H1299 cells (*P* < 0.01, Fig. 3C).

### Effect of inhibiting miR-4701-3p on the functions of NSCLC cells

**Fig. 4:**
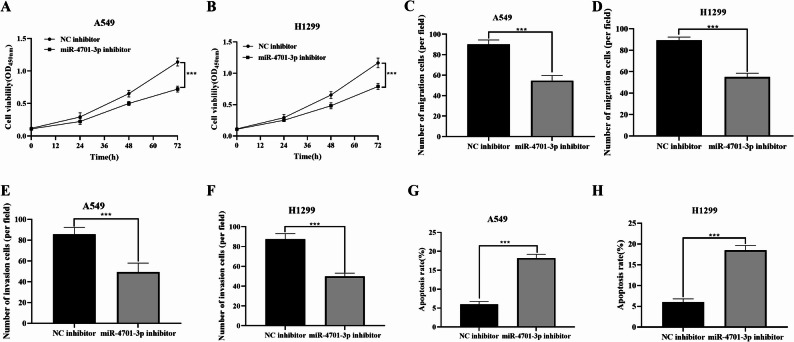
Effects of inhibiting miR-4701-3p on the proliferation, invasion, and migration of NSCLC cells (*n* = 3). **A** Proliferation capability of A549. **B** Proliferation capability of H1299. **C** Migration capability of A549. **D** Migration capability of H1299. **E** Invasion capability of A549. **F** Invasion capability of H1299. **G** Apoptosis rate of A549. **H** Apoptosis rate of H1299. Data are presented as mean ± sd. Statistical significance was analyzed using Student’s t-test. ***P < 0.01

CCK-8 cell experiments showed that knockdown of miR-4701-3p significantly inhibited the proliferation ability of NSCLC cells (*P* < 0.01, Fig. 4A, B). Transwell experiment results indicated that knockdown of miR-4701-3p inhibited both the migration (*P* < 0.01, Fig. 4C, D) and invasion (*P* < 0.01, Fig. 4E, F) abilities of NSCLC cells. Additionally, the knockdown of miR-4701-3p increased the apoptosis rate of NSCLC cells (*P* < 0.01, Fig. 4G, H).

### The effect of overexpressing miR-4701-3p on the functions of NSCLC cells

**Fig. 5:**
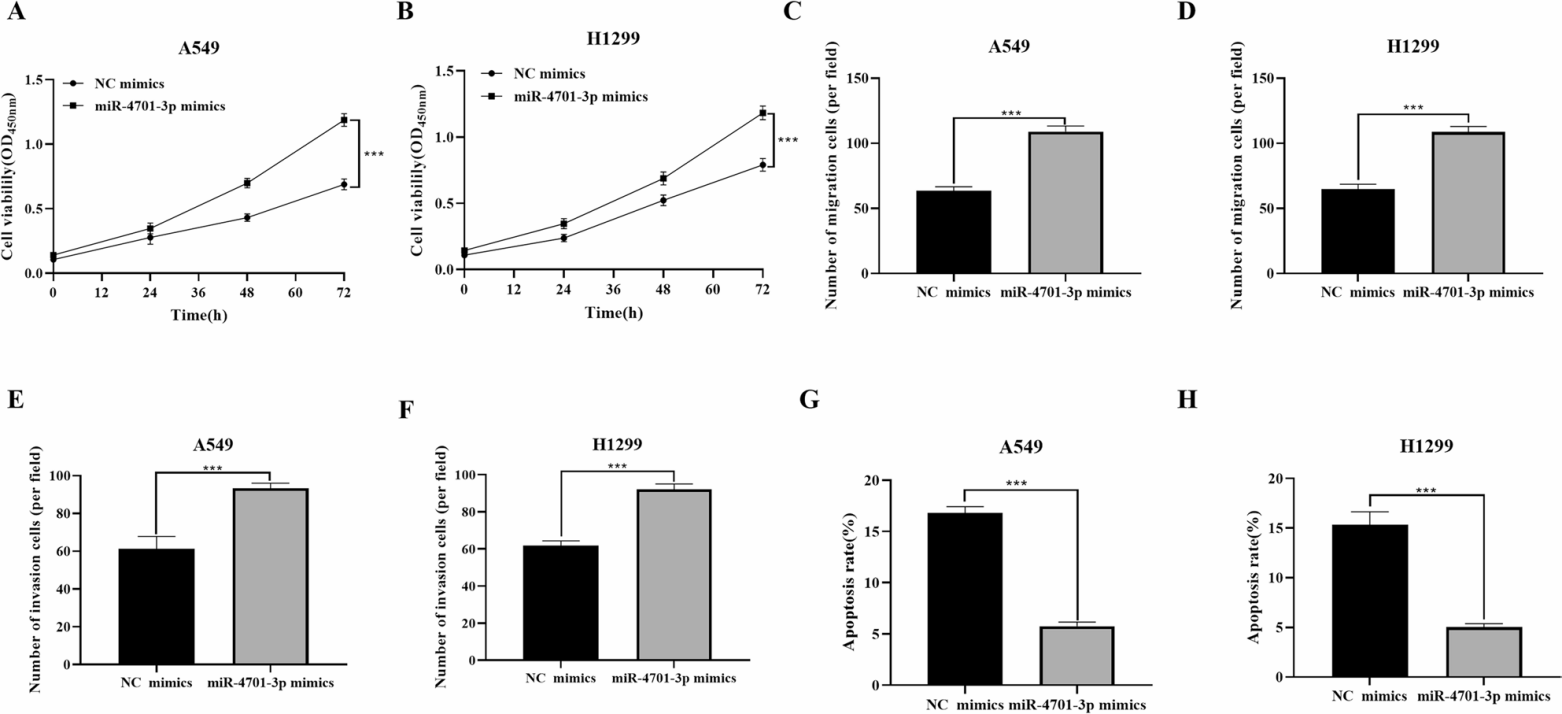
Effects of overexpressing miR-4701-3p on the proliferation, invasion, and migration of NSCLC cells (*n* = 3). **A** Proliferation capability of A549. **B** Proliferation capability of H1299. **C** Migration capability of A549. **D** Migration capability of H1299. **E** Invasion capability of A549. **F** Invasion capability of H1299. **G** Apoptosis rate of A549. **H** Apoptosis rate of H1299. Data are presented as mean ± sd. Statistical significance was analyzed using Student’s t-test. ***P < 0.01

Overexpression of miR-4701-3p significantly enhanced the proliferation ability of NSCLC cells (*P* < 0.01, Fig. 5A, B). Overexpression of miR-4701-3p promoted both the migration (*P* < 0.01, Fig. 5C, D) and invasion (*P* < 0.01, Fig. 5E, F) capabilities of NSCLC cells. Additionally, the overexpression of miR-4701-3p reduced the apoptosis rate of NSCLC cells (*P* < 0.01, Fig. 5G, H).

## Discussion

Lung cancer is characterized by rapid progression, strong invasiveness, and a high propensity for metastasis. At the time of diagnosis, most patients already exhibit distant metastases or locally invasive lymph node involvement, which contributes significantly to the poor overall prognosis [12]. Unfortunately, there is still a lack of effective indicators for assessing the long-term prognosis of lung cancer patients in clinical practice. Previous studies have shown that tumor size, LNM, pathological stage, and TNM stage are all important factors affecting the 5-year survival rate of patients after surgery [13]. The relationship between miRNAs and tumor progression remains incompletely understood. Therefore, identifying oncogenes or tumor suppressor genes involved in the initiation and progression of lung cancer, and elucidating their functions, may provide critical insights into the molecular mechanisms underlying this disease [2].

Research has demonstrated that miRNAs play pivotal roles in the initiation and progression of various malignant tumors, including gastric cancer and papillary thyroid carcinoma [14, 15]. The development of NSCLC are the results of comprehensive regulation by multiple factors. Numerous studies have indicated that miRNAs exhibit unique expression patterns and biological functions in NSCLC and can promote or inhibit its progression at different stages [16–18]. In-depth investigation of miRNAs provides a foundation for the prevention, diagnosis, and treatment of NSCLC. For example, in NSCLC, the lncRNA RMRP promotes tumor growth and progression by repressing miR-1-3p [19]. Additionally, studies have reported that miR-30e, miR-93, miR-205, and miR-221 are upregulated in squamous cell carcinoma (SCC), whereas let-7e, miR-29c, and miR-125a-5p are increased in lung adenocarcinoma (AD) [20]. This is consistent with our research findings, which revealed that miR-4701-3p is significantly increased in NSCLC tissues compared with adjacent normal tissues.

miRNA holds significant value in predicting the prognosis of NSCLC. Surgical resection continues to be the definitive therapeutic approach, yet patient survival rates remain compromised due to disease recurrence, which can be indicated by miRNA expression levels. Prior research has demonstrated that elevated miR-19b expression in NSCLC tissues correlates with advanced TNM stages, LNM, and decreased survival rates [21]. The results of this study align well with these observations. In this study, the relationship between miR-4701-3p and the clinicopathological characteristics of NSCLC patients was analyzed using chi-square tests. The results revealed that miR-4701-3p is closely related to higher TNM stage and LNM. This suggests that miR-4701-3p may be associated with the proliferation and metastasis of NSCLC, indicating that miR-4701-3p may be involved in the malignant progression of lung cancer, such as invasion and migration, and can reflect the severity of the disease in NSCLC patients. Sanforenzo and colleagues distinguished two distinct plasma miRNA signatures predictive of disease-free survival in resectable NSCLC. Specifically, one signature was associated with adenocarcinoma, characterized by elevated levels of miR-155-5p and miR-223-3p, along with decreased miR-126-3p expression. The other signature was linked to squamous cell carcinoma, marked by high miR-20a-5p expression and low levels of both miR-152-3p and miR-199a-5p [22]. Numerous studies have demonstrated the critical prognostic significance of TNM stage and LNM in NSCLC [23, 24]. This study also confirms these findings, as Cox proportional hazards analysis indicated that TNM staging, LNM, and miR-4701-3 are all risk factors influencing the survival prognosis of lung cancer patients. These findings suggest that miR-4701-3p may function as an oncogene in NSCLC and has potential value as a prognostic biomarker to guide clinical diagnosis and treatment decisions.

miRNAs participate in various physiological processes, including cell proliferation and signaling pathways of different target proteins [25]. Metastasis and invasion are important biological characteristics of NSCLC and are the primary factors determining poor prognosis [26]. Downregulated miR-497-5p in NSCLC can inhibit its progression by suppressing the expression of the SOX5 gene [27]. miR-600 can inhibit the proliferation and migration of lung cancer [28]. The findings suggest that miRNAs can function as either oncogenes or tumor suppressors, regulating tumorigenesis through complex downstream mechanisms. Previous studies have found that miR-4701-3p can enhance the viability of colon cancer cells and inhibit cell apoptosis [10]. Our study’s observations align with these findings, which revealed that knockdown of miR-4701-3p can significantly inhibit the proliferation, migration, and invasion abilities of NSCLC cells and promote their apoptosis. Overexpression of miR-4701-3p can significantly promote the proliferation, migration, and invasion abilities of NSCLC cells while inhibiting their apoptosis. The study demonstrates that miR-4701-3p can influence the malignant biological behaviors of NSCLC cells. This study not only deepens our understanding of the biological functions of miR-4701-3p but also provides important experimental evidence supporting its potential as a molecular biomarker for evaluating long-term prognosis in NSCLC patients.

Nonetheless, the precise molecular mechanism underlying the role of miR-4701-3p in the current study remains incompletely understood. In exploring potential downstream mechanisms, bioinformatics prediction suggests that TOB2 may be a putative target of miR-4701-3p [29]. TOB2, a member of the anti-proliferative TOB/BTG family, has been implicated in regulating cell growth, apoptosis, and differentiation in several cancer types and cardiovascular conditions [30, 31]. Future studies, including dual-luciferase reporter assays and functional rescue experiments, are warranted to confirm whether TOB2 or other predicted targets mediate the biological functions of miR-4701-3p in NSCLC. While this study demonstrates the clinical relevance and functional effects of miR-4701-3p in NSCLC, the investigation remains primarily phenomenological. Given the complexity of gene regulatory networks, a single miRNA can modulate multiple downstream targets. Future studies will incorporate bioinformatics prediction, functional enrichment analysis, and dual-luciferase reporter assays to identify the direct target genes and key signaling pathways regulated by miR-4701-3p, thereby providing deeper insight into its molecular role in NSCLC progression. These investigations will enhance our comprehensive knowledge of the biological roles played by miR-4701-3p and its potential application value in NSCLC. Given the stability of miRNAs in serum and other body fluids, miR-4701-3p holds considerable promise as a circulating biomarker for NSCLC. However, clinical translation will require overcoming challenges related to miRNA delivery, stability, and off-target effects. Although this study provides strong evidence supporting the clinical potential of miR-4701-3p, our conclusions are based on in vitro findings and limited patient samples. Large-scale clinical studies are needed to verify its diagnostic and prognostic accuracy, while preclinical models are required to assess the safety and efficacy of miR-4701-3p-based therapeutic interventions.

## Conclusion

In summary, the research conducted in this study revealed that miR-4701-3p is significantly upregulated in NSCLC and has certain predictive value for poor patient prognosis. It may be involved in malignant behaviors of NSCLC cells, such as proliferation, migration, and invasion.

## Data Availability

All data generated or analyzed during this study are included in this article and its supplementary material files. Further enquiries can be directed to the corresponding author.
